# Metabolomics Deciphering the Potential Biomarkers of Hengqing I Prescription against Vascular Dementia

**DOI:** 10.1155/2022/1636145

**Published:** 2022-03-30

**Authors:** Shengxi Meng, Shaopeng Li, Huize Chen, Chujun Deng, Zeyu Meng, Yimo Wang

**Affiliations:** ^1^Department of Traditional Chinese Medicine, Shanghai Jiao Tong University Affiliated Sixth People's Hospital, Shanghai 200233, China; ^2^Shanghai Sensichip Infotech, Shanghai 200331, China; ^3^The Second Clinical Medical College, Heilongjiang University of Chinese Medicine, Harbin 150040, China

## Abstract

With the aging of population, vascular dementia (VaD) seriously threatens people's health and quality of life. It is of great significance to explore biomarkers of VaD from the perspective of metabolomics and traditional Chinese medicine (TCM). Therefore, VaD was divided into kidney deficiency and blood stasis syndrome (KDBS) and non-KDBS according to TCM. Then, some patients received the treatment of Hengqing I (HQI) prescription. The urine of six groups (VaD group, normal group, KDBS group, non-KDBS group, HQI group, and control group) was detected on LC-MS/MS. Multivariate statistical analysis showed that the metabolic profiles of the three comparisons were significantly different. The top analysis-ready molecules of downregulated histamine and upregulated biotin, methionine, pantothenic acid, SAH, histidine, and kaempferol may be the most related metabolites. These putative biomarkers play an important role in the regulation of key metabolic processes linked to VaD. Additionally, pathway analysis showed aminoacyl-tRNA biosynthesis, and amino acids metabolic pathways were highly correlated with the occurrence of VaD. In this present paper, vitamins, amino acids, and their derivatives were selected as the basis for VaD diagnosis and treatment monitoring, and the significance of TCM classification and Hengqing I prescription in the treatment of VaD was discussed.

## 1. Introduction

As the second most common type of dementia in the world, vascular dementia (VaD) is caused by multiple ischemic foci in the brain [1–3]. To date, people suffering from VaD increased around the world, especially the elderly. With the ageing of society, Cylus and Tayara proposed that if older workers are healthy, ageing will not result in economic decline [4]. However, there is no effective drug for the treatment of VaD, and the clinical diagnosis of VaD is not so easy [5, 6]. This poses a great threat to the health and quality of life of the elderly [7]. Recently, many studies focused on the biomarkers of VaD [8, 9]. Liu found that lipids ceramides and cholesterol ester were abnormal in VaD patients compared with the healthy group [10]. In addition, traditional Chinese medicine (TCM) YGS significantly improved 2-VO (two-vessel occlusion) induced cognitive impairment and alleviated hippocampal histopathological abnormalities in rat [2]. Although biomarkers of VaD have been extensively studied, the pathophysiological mechanism regulating the VaD severity and clinical phenotype is not completely clear. Therefore, the specific markers of VaD are very important for the diagnosis and treatment of VaD. Moreover, these studies mainly focused on blood biomarkers in VaD and rarely involve the study on urine metabolites. Finding biomarkers from urine could provide a new perspective to study VaD.

Facing the dilemma that there are still no drugs for VaD, TCM is a huge resource for the explanation on VaD. TCM plays a more and more important role in the prevention and treatment of VaD and gradually shows a great application prospect [2, 11, 12]. However, the use of TCM in the treatment of VaD still lacks the corresponding theoretical basis. We consider that the classification of VaD according to TCM syndrome differentiation can provide medication basis. VaD can be divided into kidney deficiency and blood stasis syndrome (KDBS) and non-KDBS according to TCM syndrome differentiation [13], and there is no relevant research on the difference of metabolomics between them. Studying the metabolomic differences between them has great theoretical significance for the treatment of VaD by TCM. Hengqing I, a TCM compound, is an effective prescription for the treatment of VaD summarized by our research group [14, 15]. One dose of Hengqing I is composed of ten medicinal herbs: *Alpinia oxyphylla* Miq. (30 g), *Cuscuta chinensis* Lam. (15 g), *Ligusticum chuanxiong* Hort. (15 g), *Acorus tatarinowii* Schott (20 g), *Rehmannia glutinosa* Libosch. (15 g), *Eucommia ulmoides* Oliv. (15 g), *Pheretima aspergillum* (E.Perrier) (10 g powder), *Curcuma longa* L. (15 g), *Gastrodia elata* Bl. (10 g), and *Uncaria macrophylla* Wall. (10 g) with simmer in water. Hengqing I can significantly improve the cognitive function of patients with VaD, reduce the levels of serum triacylglycerol, total cholesterol, and low-density lipoprotein cholesterol, increase the level of Mini-Mental State Examination (MMSE), and improve Hasegawa Dementia Scale (HDS) and Activity of Daily Living Scale (ADL) [14]. However, due to the complicated chemical composition and the unknown synergistic actions from these multiple components, the molecular mechanism underlying the protective effects of Hengqing I on VaD still remains unclear. Therefore, the use of scale to measure the therapeutic effects of VaD still lacks objective basis. Therefore, it is necessary to study the effects of Hengqing I on VaD and the TCM classification of VaD by metabolomics.

In recent years, metabolomics research has made great progress. The efficiency and reliability of metabolite detection have been improved [16, 17]. It is a powerful way to study VaD pathogenic mechanism and explore VaD biomarkers. In order to provide a new understanding of the pathogenesis of VaD from urine metabolites, this study observed the metabolomic differences between VAD and healthy individuals. Then, the biomarkers of VaD of taking Hengqing I were screened, and a new clinical research strategy of TCM in the treatment of VaD was explored.

## 2. Materials and Methods

### 2.1. Study Population and Urine Samples Collection

This study recruited 90 VaD patients as VaD group and 30 healthy volunteers as normal group. After exclusion and abscission criteria, 87 VaD patients were retained. According to TCM syndrome differentiation, VaD group was divided into KDBS group (59 patients) and non-KDBS group (28 patients). Urine samples from normal cases and VaD patients (including KDBS group and non-KDBS group) were collected. KDBS cases were further randomly divided into HQI group (29 patients) and control group (30 patients). HQI group patients took TCM prescription Hengqing I once a day for five days per week, while control group patients took equivalent dummy pills. On this basis, VaD patients with hypertension and diabetes should be given corresponding to antihypertensive treatment and hypoglycemic treatment. After eight weeks, urine samples were collected from HQI group and control group. Urine samples were stored at −80°C, and then the metabolites in urine samples were detected. This study was approved by Shanghai Sixth People's Hospital Ethics Committee, and ethics approval number is 2019-012.

### 2.2. Clinical Cognitive Assessment for VaD Patients

In order to evaluate the changes of cognitive level of VaD patients after taking Hengqing I, we tested the cognitive scale of HQI group and control group before treatment and after treatment eight weeks. Cognitive level tests included MMSE score, HDS score, and ADL score. MMSE score ranges from 0 to 30, including language understanding, language expression, reading comprehension, time orientation, speech expression, attention, drawing, short-term memory, and computing ability. The lower the score, the more serious the cognitive impairment. The HDS score ranges from 0 to 32.5. The score above 31 is normal, the score of 22 to 30.5 is lower than normal, the score of 10.5 to 21.5 is predementia, and the score below 10 is dementia. The full score of ADL is 100. The lower score, the worse the living ability.

### 2.3. Pretreatment of Urine Samples

The samples were unfrozen on ice, and 100 *μ*L was transferred to 1.5 mL centrifugal tube. The solution (300 *μ*L methanol) and internal standard (10 *μ*L) were added into samples. Internal standard consists of 2.8 mg/mL DL-o-chlorophenylalanine with 2.8 mg/mL lysophosphatidylcholine (12 : 0). The samples were shaken 30 seconds and placed one hour at 4°C for dissolving samples. After 15 min centrifugation (12000 rpm/min), all supernatant was taken into a new tube and placed one hour at −40°C. Centrifugation was done one more time at 4°C. The supernatant (200 *μ*L) was transferred into small bottle for LC-MS/MS loading. In addition, 5 *μ*L supernatant of each sample was mixed as 14 quality control (QC) samples to ensure the stability of LC-MS/MS data.

### 2.4. LC-MS/MS Parameters and Liquid Phase Composition

UHPLC (Ultra High Performance Liquid Chromatography LC-30A TOF5600+ (AB SCIEX)) was used to separate samples, and XBridge Amide column was used to separate urine metabolites [18, 19]. Liquid phase A consists of ultrapure water with 10 mM ammonium formate and 0.1% (v/v) formic acid. Liquid phase B was chromatographic acetonitrile. Elution gradients of liquid B were 95%B (0.5 min), 95%B–65%B (0.5–10 min), 65%B–40%B (10–14 min), 40%B–40%B (14–16 min), 40%B–95%B (16–16.1 min), and 95%B–95%B (16.1–20 min). Elution rate was 0.8 mL/min, injection volume was 4 *μ*L, and column temperature was 40°C.

Mass spectrometer (Triple TOF5600+, AB SCIEX, USA) with electron spray ionization was used to detect fragment ion of metabolites. Both sheath gas and aux gas were nitrogen, and the flow rates were 35 arb and 15 arb, respectively. Mass spectrometer scan ranges from 100 to 1000 m/*z*. Electron spray voltage was 4.2 KV, and ion transmission capillary temperature was 350°C. The data were collected by high resolution Fourier transform mode. The resolution of MS1 was 60000, and MS2 was 15000. MS2 data were collected by data-dependent analyst mode. Dynamic exclusion time was 15 s. Collision mode was higher energy collisional dissociation. Collisional energy was 20%, 40%, and 60% basis on different metabolites. Isolation width was 3 Da, and activation time was 30 ms.

### 2.5. Data Analysis

Raw data were converted to ABF format by AbfConverter 4.0.0. MS-DIAL was used for peak extraction, peak contrast, peak filter, fill gap, and material identification. Then, data were normalized to two-dimensional data matrix (Excel table). Two-dimensional data consist of retention time, m/*z*, metabolites peak area, and other information. The data matrix after pretreatment was multivariate statistical analysis by SIMCA-P 14.0 (Umetrics AB, Umea, Sweden) [20]. If the data conformed to normal distribution, the data could be expressed as mean and standard deviation, otherwise as median and quartile. If comparison between the two groups accorded with normal distribution and homogeneity of variance, the data were analyzed by Student's test, otherwise else by Mann-Whitney U nonparametric test. If *P* value is less than 0.05, the differences are considered to be significant. The data analysis was completed by SPSS 13.0 statistical software.

## 3. Results

### 3.1. Effect of Hengqing I on Cognitive Level of VaD Patients

After treatment, MMSE score, HDS score, and ADL score in the control group increased, but there was no significant difference compared with those before treatment (*P* > 0.05). MMSE score, HDS score, and ADL score in the HQI group were significantly higher than the control group (*P* < 0.05, Table 1). These results showed that Hengqing I can significantly improve the cognitive level of patients with VaD. In order to understand what components of Hengqing I against VaD, we performed subsequent metabolomic analysis of urine samples.

### 3.2. Multivariate Analysis of LC-MS/MS Results

**Figure 1:**
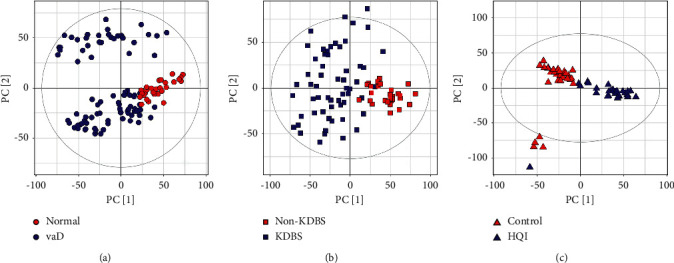
The PCA score plots of VaD ratio normal (a), KDBS ratio non-KDBS (b), and HQI ratio control group (c).

The multivariate analysis was used to measure the changes in the urine metabolic profiles of six groups (VaD group, normal group, KDBS group, non-KDBS group, HQI group, and control group) [21]. The normal group was healthy individuals without VaD. The control group was VaD patients who did not take Hengqing I. PCA analysis is an unsupervised model analysis method, which can classify data according to their similarity and reflect the differences of overall metabolic profile between two groups [22]. In Figure 1, there are obvious clusters that are consistent with the group in three comparisons, which illustrates that there are significant metabolic differences between the two groups. Meanwhile, the relative standard deviation of the peak area of QC is 16.05%, indicating that the data has good stability.

**Figure 2:**
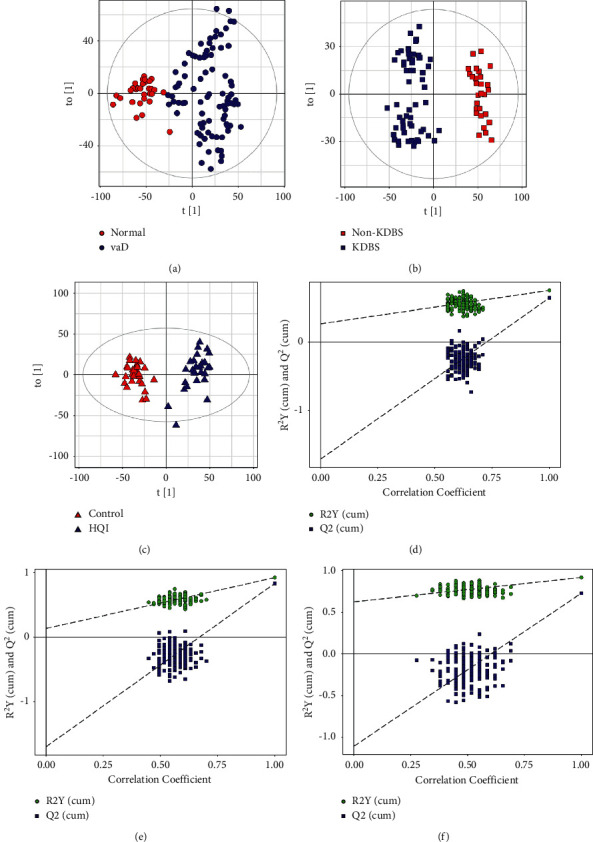
The OPLS-DA score plots of VaD ratio normal (a), KDBS ratio non-KDBS (b), and HQI ratio control group (c). (d–f) OPLS-DA permutation plots.

In addition to principal component analysis, the OPLS-DA (orthogonal partial least squares-discriminant analysis) was further used to analyze the data [23]. OPLS-DA can determine which metabolite variables cause differences between groups and obtain the contribution rate of different substances for each group, which makes it simpler to find the main variables. The OPLS-DA scores are presented in Figure 2. VaD and KDBS samples could be clearly distinguished from normal and non-KDBS samples, and HQI and control samples could clearly be distinguished. As shown in Figures 2(d)–2(f), the models possess satisfactory fit, which indicates that there are discriminations of the urine metabolomics signature between groups.

### 3.3. Differential Metabolites of VaD Ratio Normal, KDBS Ratio Non-KDBS, and HQI Ratio Control

The urine metabolites from each group were examined. Variance importance projection (VIP) scores demonstrated the interrelated significance of each metabolite for distinguishing two groups. VIP scores provide a method to understand the importance of metabolites better, as higher VIP scores indicate a greater discrimination contribution. VIP scores greater than 1 for the first principal component of the OPLS-DA model and combined with the *P*-value (threshold value of 0.05) of Student's *t*-test (*T*-test) can be used to determine the differential metabolites. When VaD cases were compared with the normal group cases, 90 metabolites were significantly altered (59 upregulated and 31 downregulated, Table 2). The top ten most altered differential metabolites were biotin, N-acetyl-L-leucine, cis-4-hydroxy-D-proline, 1,7-dimethyluric acid, 4-hydroxy-6-methyl-2-pyrone, H-Pro-Hyp-OH, decanoylcarnitine, 5-methyluridine, and 5-aminolevulinic. These upregulated differential metabolites are highly correlated with VaD. The identification criteria of metabolites are that mass tolerance is 10 ppm and match factor threshold is 60.

Moreover, there were 86 different metabolites in the urine samples of KDBS cases compared with non-KDBS cases, including most altered pantothenic acid, norleucine, biotin, N-cinnamoylglycine, pyroglutamic acid, N-acetyl-L-leucine, 5-aminolevulinic acid, hexamethylene bisacetamide, cis-4-hydroxy-D-proline, and N-acetylputrescine (*P* < 0.05, Table 3). Among them, biotin, cis-4-hydroxy-D-proline, N-acetyl-L-leucine, 5-aminolevulinic acid, and H-Pro-Hyp-OH also appeared in VaD ratio normal comparison. What is more, there are some metabolic differences in Table 2. For example, pantothenic acid, norleucine, N-cinnamoylglycine, and pyroglutamic acid are significantly upregulated (logFC >3), which shows that KDBS type VaD patients not only have the metabolites of common VaD cases, but also have new significantly upregulated differential metabolites, compared with non-KDBS patients. This refers to that the classification of VaD through TCM syndrome differentiation is true and reliable. At the same time, these significantly upregulated substances can be used as the biomarkers of VaD from kidney deficiency and blood stasis. A total of 84 metabolites were significantly distinguishable between HQI group and control group (42 upregulated and 42 downregulated, Table 4), including most altered histamine, hexamethylene bisacetamide, dopamine, decanoylcarnitine, xanthurenic acid, N-acetylleucine, 4-methylpyrimidine, and 1-methylnicotinamide. This comparison revealed a substantial number of novel divergent metabolites, which might indicate alterations in the pathogenic process of VaD. These novel differential metabolites might be the reason why Hengqing I treatment improves VaD patients' cognitive abilities.

### 3.4. Pathway Analysis of Three Comparisons

**Figure 3:**
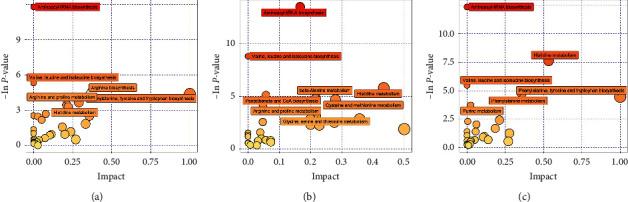
The metabolic pathway map of VaD ratio normal (a), KDBS ratio non-KDBS (b), and HQI ratio control group (c). Pathway names are displayed with *P* < 0.05.

We located the above differential metabolites to metabolic pathways by MetaboAnalyst 5.0 website [24]. The found pathways were screened with *P*-value < 0.05 and impact value ≥ 0. The smaller the *P*-value, the more reliable the metabolic pathway. The impact value represents the influence of metabolites on the metabolic pathway. In Figure 3, the three comparisons located 6, 8, and 6 pathways, respectively. In the VaD ratio normal, we found aminoacyl-tRNA biosynthesis, valine, leucine and isoleucine biosynthesis, arginine biosynthesis, arginine and proline metabolism, phenylalanine, tyrosine and tryptophan biosynthesis, and histidine metabolism pathways. In the second comparison, the beta-alanine metabolism, pantothenate and CoA biosynthesis, cysteine and methionine metabolism and glycine, serine, and threonine metabolism pathways were unique. Meanwhile, the phenylalanine metabolism and purine metabolism were specific in HQI ratio control.

## 4. Discussion

In pathway analysis, it should be noted that the aminoacyl-tRNA biosynthesis pathway appeared in three comparisons. And this pathway mapped the greatest number of differential metabolites, indicating that this pathway actively participates in the pathogenesis and treatment of VaD, which is consistent with the research results made by Hosoki, Tanaka, and Ihara. However, the impact value of this pathway was less than most pathways in three comparisons. This showed that the influence of aminoacyl-tRNA biosynthesis had a weaker effect on VaD than other pathways. In addition, there were a large number of amino acid metabolic pathways in the three comparisons, such as valine biosynthesis, histidine metabolism, and methionine metabolism. Amino acids synthesis and metabolism could affect the physiological phenomena related to cerebral vessels and finally affect the cognitive function of the brain, which will be an important idea for us to screen biomarkers in differential metabolites.

Therefore, we screened biomarkers according to the metabolic pathways and the fold change of differential metabolites. In comparison of normal group, biotin had max fold change in VaD (logFC was 4.10). Biotin, known as vitamin B7 or H, is an organic heterobicyclic compound, and it presents in minute amounts in every living cell [25, 26]. Cooper found that the biotin deficiency occurs in dementia patients compared with healthy cases [27]. We found that the contents of biotin in urine of VaD and KDBS type VaD patients were both upregulated significantly, which is intuitively inconsistent with Cooper's conclusion. It was speculated that VaD patients cannot make good use of biotin, thus resulting in a large amount of biotin excretion through urine. However, this hypothesis needs to be proved by subsequent studies on the content of biotin in blood and brain. In amino acids, isoleucine also significantly upregulated in VaD and HQI group. Isoleucine was mapped at valine, leucine, and isoleucine biosynthesis in pathway analysis. These three amino acids were BCAA (branched chain amino acid). The metabolic abnormality of BCAA is mainly related to diabetes [28], but the relationship between BCAA and VaD has not been reported yet. And valine, leucine, and isoleucine biosynthesis had small impact value in three comparisons. This showed that it had a weaker effect on VaD than other pathways, which was similar to aminoacyl-tRNA biosynthesis. In addition, methionine was upregulated in VaD and KDBS type VaD patients compared with another group. Studies on feeding rodents high levels of methionine have shown that methionine could promote atherosclerotic plaques [29, 30]. A similar study in Finnish men showed the same effect [31]. It is reasonable to suspect that the increase of methionine could lead to the blockage of cerebral blood vessels and then cerebral ischemia.

The metabolomic differences between KDBS and non-KDBS VaD were also studied. To the best of our knowledge, this is the first study on the metabolite profiles in VaD with KDBS and non-KDBS. In addition to the above biotin and methionine, new metabolic differential substances pantothenic acid and pyroglutamic acid were upregulated in KDBS group. Pantothenic Acid (Vitamin B5) is a water-soluble vitamin ubiquitously in plants and animal tissues with antioxidant property. Vitamin B5 is a growth factor and it is essential for various metabolic functions, including the synthesis of cholesterol, lipids, neurotransmitters, and hemoglobin. Xu revealed that severe vitamin B5 brain deficiency was common in AD patients, especially in areas with severe brain damage [32]. From research, abnormal pantothenic acid levels in dementia patients were also found [27]. In this study, same as the biotin situation, pantothenic acid was upregulated in KDBS group. Pyroglutamic acid (5-oxoproline) is a cyclized derivative of L-glutamic acid. Elevated blood levels are related to glutamine or glutathione metabolism. At present, there is no report on pyrolysine and VaD related diseases. The relationship between pyroglutamic acid and VaD needs more research. S-Adenosyl-L-homocysteine (SAH) was upregulated in KDBS urine. SAH is almost the product of all methylation reactions that involve S-adenosylmethionine (SAM) as the methyl donor. The disturbances in the transmethylation pathway indicated by abnormal SAH, SAM, or their ratio have been reported in many neurodegenerative diseases, such as dementia, depression, and Parkinson's disease [33, 34]. Cervellati also observed a trend toward elevation of homocysteine in VaD compared with control [35]. This displays that cerebrovascular blockage by homocysteine is the cause of KDBS type VaD, which is similar to the significant upregulation of methionine. Moreover, in this comparison, we found a unique methionine synthesis pathway. The two comparisons (VaD ratio normal, KDBS ratio non-KDBS) had the same metabolites and specific metabolites. However, they both suggested that the blockage of blood vessels could lead to cerebral ischemia and the increase of vitamin B in urine. These differential metabolites proved that VaD can be classified through TCM syndrome differentiation, and then the corresponding treatment can be carried out through TCM.

Compared with the control group, histamine decreased significantly after VaD patients took Hengqing I. Histamine is an amine derived by enzymatic decarboxylation of histidine. Some studies showed that histamine is regarded as a vasodilator [36, 37]. This may be that, when the cerebral focus of VaD patients is ischemic, their body would induce a large amount of histamine to expand blood vessels to make up for blood supply. Hengqing I prescription could improve the blood supply to the brain, so the contents of histamine in urine decrease significantly. At the same time, histidine was significantly upregulated, which may downregulate histamine, thus leading to the accumulation of histidine. It is worth noting that histidine metabolism appeared in the three comparisons. But the impact of histidine metabolism in HQI ratio control was max (impact > 0.5). This showed that taking Hengqing I prescription had a great effect on the histidine metabolism. Moreover, after taking Hengqing I prescription, kaempferol, a tetrahydroxyflavone from plant, was significantly upregulated. Kaempferol acts as an antioxidant by reducing oxidative stress. According to previous studies, kaempferol may protect the brain of the elderly, and kaempferol can prevent hippocampal injury and memory impairment caused by cadmium chloride [38]. Kaempferol in ginseng also has certain effects on the treatment of Alzheimer's disease [39]. And Kaempferol did not appear in the previous two comparisons. It can be inferred that kaempferol in the patient's urine was ingested through Hengqing I prescription.

**Figure 4:**
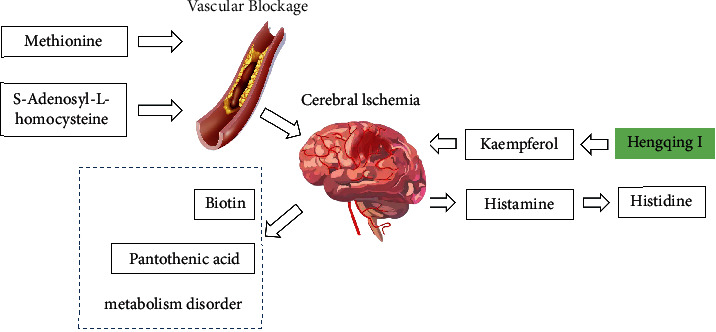
Relationship between different metabolites and VaD and biomarkers of Hengqing I against VaD.

Based on the above discussion, we regard that the methionine and S-Adenosyl-L-homocysteine caused blockage of blood vessels in the brain and then led to cerebral ischemia. Cerebral ischemia can cause metabolic disorder of vitamin B (biotin and pantothenic acid). Kaempferol in Hengqing I can relieve cerebral ischemia in VaD patients, so that a large amount of histamine is not required to dilate blood vessels, which reduces the content of histamine and accumulates histidine (Figure 4). Histamine, histidine, and kaempferol could be as biomarkers of Hengqing I against VaD.

## 5. Conclusions

Our work suggested that urine metabolomics exhibits considerable potential in elucidating the response mechanisms of VaD cases. The metabolic profiles of VaD ratio normal, KDBS ratio non-KDBS, and HQI ratio control were significantly different. Then, over 90 differential metabolites were found. Metabolic pathway analysis showed that aminoacyl-tRNA biosynthesis and amino acids metabolic pathways are highly correlated with the occurrence of vascular dementia. Among differential metabolites, biotin, methionine, pantothenic acid, and S-Adenosyl-L-homocysteine were related to cerebral ischemia. Hengqing I prescription may relieve the cerebral ischemia and lead to the downregulation of histamine and the upregulation of histidine. These metabolites deepen our understanding of the disease mechanism of VaD and provide the possibility for the effective treatment of VaD.

## Figures and Tables

**Table 1:** Effect of Hengqing I on cognitive level of VaD patients.

Group	MMSE score		HDS score		ADL score	
	Before treatment	After treatment	Before treatment	After treatment	Before treatment	After treatment
HQI	22.45 ± 3.32	28.48 ± 4.32ab	23.48 ± 3.22	30.30 ± 3.72ab	41.88 ± 9.38	76.5 ± 6.72ab
Control	23.08 ± 3.15	25.52 ± 3.39	22.36 ± 3.95	27.32 ± 4.8	43.49 ± 9.14	58.49 ± 7.33

Small “a” indicates significant difference compared with that before treatment (*P* < 0.05). Small “b” indicates significant difference compared with the control group (*P* < 0.05).

**Table 2:** Differential analysis of the metabolites in VaD and normal groups (as shown in the first 20 lines).

Name	m/*z*^1^	RT^2^ (min)	VIP	*P*-value	LogFC^3^
Biotin	245.09	5.12	1.05	1.52E-06	4.10
N-Acetyl-L-leucine	174.11	4.15	1.48	8.72E-10	3.72
cis-4-Hydroxy-D-proline	132.06	11.87	1.87	2.75E-10	2.68
4-Hydroxy-6-methyl-2-pyrone	127.04	3.15	1.60	1.21E-09	2.46
1,7-Dimethyluric acid	197.07	6.10	1.13	1.56E-04	2.33
H-Pro-Hyp-OH	229.12	11.92	1.97	1.47E-09	2.29
Decanoylcarnitine	316.25	6.53	1.79	2.44E-14	2.21
5-Methyluridine	259.09	11.17	1.60	5.52E-04	2.18
5-Aminolevulinic acid	132.06	5.00	1.08	6.75E-05	2.04
Naringenin	273.08	6.45	1.06	9.81E-03	-2.00
3-Aminoisobutyric acid	104.07	10.51	1.34	7.05E-06	1.86
Kynurenine	209.09	8.96	1.87	3.67E-10	1.82
Isoleucine	132.10	9.21	2.33	4.17E-09	1.81
6-Hydroxynicotinic acid	140.04	8.97	1.31	9.53E-04	1.80
Sorbitol	205.07	9.84	1.44	2.73E-02	-1.79
Deoxycarnitine	146.12	10.29	1.73	3.59E-07	1.77
Asp-Phe	281.10	10.84	1.74	1.09E-03	1.69
Tyrosine	182.08	10.09	1.70	3.80E-03	-1.66
Methionine	150.06	9.55	1.88	8.48E-09	1.54
Trigonelline	160.04	9.83	1.98	1.67E-05	-1.48

^1^Mass-to-charge ratio; ^2^retention time; ^3^logarithm of fold change based on two.

**Table 3:** Differential analysis of the metabolites in KDBS and non-KDBS groups (as shown in the first 20 lines).

Name	m/*z*^1^	RT^2^ (min)	VIP	*P*-value	LogFC^3^
Pantothenic acid	220.12	5.22	1.81	3.00*E* − 05	5.90
Norleucine	132.10	4.15	1.80	5.55*E* − 09	4.66
Biotin	245.09	5.12	1.77	8.15*E* − 07	4.44
N-Cinnamoylglycine	206.08	4.64	1.64	9.54*E* − 05	4.15
Pyroglutamic acid	130.05	6.29	1.50	4.77*E* − 08	3.61
N-Acetyl-L-leucine	174.11	4.15	1.90	5.87*E* − 10	3.47
5-Aminolevulinic acid	132.06	5.00	1.45	6.06*E* − 07	2.59
Hexamethylene bisacetamide	201.16	8.88	1.20	1.64*E* − 03	1.90
cis-4-Hydroxy-D-proline	132.06	11.87	1.90	1.10*E* − 07	1.88
N-Acetylputrescine	131.11	9.54	1.70	4.75*E* − 09	−1.83
3-Indoleacetic acid	176.07	6.68	1.88	2.94*E* − 04	−1.81
H-Pro-Hyp-OH	229.12	11.92	1.73	3.73*E* − 07	1.75
Phosphocholine	184.07	12.71	1.39	1.12*E* − 02	1.50
S-Adenosyl-L-homocysteine	385.13	12.16	2.02	1.92*E* − 05	1.49
5-Methylcytidine	258.11	8.56	1.08	3.83*E* − 04	1.48
D-Maltose	343.12	12.34	1.59	2.91*E* − 03	1.44
Acetylhomoserine	162.07	7.21	1.60	1.60*E* − 06	−1.42
Ecgonine	186.11	4.31	1.46	2.62*E* − 03	−1.35
Hippuric acid	180.07	4.44	1.76	2.45*E* − 06	−1.35
L-Citrulline	176.10	12.21	1.75	3.93*E* − 03	1.32

^1^Mass-to-charge ratio; ^2^retention time; ^3^logarithm of fold change based on two.

**Table 4:** Differential analysis of the metabolites in HQI and control groups (as shown in the first 20 lines).

Name	m/*z*^1^	RT^2^ (min)	VIP	*P*-value	LogFC^3^
Histamine	1act12.09	9.14	1.55	8.58*E* − 03	−3.11
Hexamethylene bisacetamide	201.16	8.88	2.43	2.48*E* − 06	−3.03
Dopamine	154.10	9.15	1.21	8.12*E* − 03	−2.84
Decanoylcarnitine	316.25	6.53	2.20	2.04*E* − 07	2.54
Xanthurenic acid	206.04	7.44	1.96	2.17*E* − 04	−2.44
N-Acetyl-L-leucine	174.11	4.15	1.77	6.18*E* − 03	2.42
4-Methylpyrimidine	95.06	9.14	1.42	9.51*E* − 03	−2.36
1-Methylnicotinamide	137.07	9.37	2.16	2.67*E* − 09	−2.08
5-Methyluridine	259.09	11.17	1.64	1.73*E* − 02	2.06
Kynurenine	209.09	8.96	2.14	2.03*E* − 06	2.04
H-Pro-Hyp-OH	229.12	11.92	1.78	1.24*E* − 05	1.83
Methionine	150.06	9.55	2.24	3.08*E* − 06	1.82
Methylguanidine	74.07	8.80	1.30	3.91*E* − 03	1.76
Isoleucine	132.10	9.21	2.33	1.33*E* − 10	1.72
5-Methylcytosine	126.07	8.26	1.42	1.10*E* − 04	1.63
Asp-Phe	281.10	10.84	1.86	7.88*E* − 03	1.56
Propionylcarnitine	218.14	8.81	1.48	2.53*E* − 03	1.54
Tyrosine	182.08	10.09	1.68	3.20*E* − 02	−1.53
Kaempferol	287.05	1.52	1.07	3.42*E* − 02	1.46
Histidine	156.08	13.74	1.52	3.11*E* − 04	1.24

^1^Mass-to-charge ratio; ^2^retention time; ^3^logarithm of fold change based on two.

## Data Availability

The data used to support the findings of this study are included within the article.

## References

[1] Gorelick P. B., Scuteri A., Black S. E. (2011). Vascular contributions to cognitive impairment and dementia. *Stroke*.

[2] Liao W., Xue Z., Wang X. (2020). Metabolic profiling deciphering the potential targets of Yi-Gan San against vascular dementia in rat. *Brain Research*.

[3] Shih A. Y., Blinder P., Tsai P. S. (2013). The smallest stroke: occlusion of one penetrating vessel leads to infarction and a cognitive deficit. *Nature Neuroscience*.

[4] Cylus J., Tayara L. A. (2021). Health, an ageing labour force, and the economy: does health moderate the relationship between population age-structure and economic growth?. *Social Science & Medicine*.

[5] Engelborghs S., Le Bastard N. (2012). The role of CSF biomarkers in the diagnostic work-up of mixed vascular-degenerative dementia. *Journal of the Neurological Sciences*.

[6] Nagata K., Saito H., Ueno T. (2007). Clinical diagnosis of vascular dementia. *Journal of the Neurological Sciences*.

[7] Kirk A., Kushneriuk M., Karunanayake C., Morgan D., O’Conell M. (2021). Quality of life compared in mild cognitive impairment, Alzheimer’s, frontotemporal, Lewy body, and vascular dementia. *Journal of the Neurological Sciences*.

[8] Hosoki S., Tanaka T., Ihara M. (2021). Diagnostic and prognostic blood biomarkers in vascular dementia: from the viewpoint of ischemic stroke. *Neurochemistry International*.

[9] Miceli V., Russelli G., Iannolo G. (2020). Role of non-coding RNAs in age-related vascular cognitive impairment: an overview on diagnostic/prognostic value in Vascular Dementia and Vascular Parkinsonism. *Mechanisms of Ageing and Development*.

[10] Liu Y., Chan D. K. Y., Thalamuthu A. (2020). Plasma lipidomic biomarker analysis reveals distinct lipid changes in vascular dementia. *Computational and Structural Biotechnology Journal*.

[11] Tian D., Gao Q., Lin J. (2021). Uncovering the mechanism of the Shenzhi Jiannao formula against vascular dementia using a combined network pharmacology approach and molecular biology. *Phytomedicine*.

[12] Zhao D., Yi Y., He Q., Wang S., Yang K., Ge J. (2021). Exploring the regulatory mechanism of Nao Tai Fang on vascular Dementia’s biological network based on cheminformatics and transcriptomics strategy. *Journal of Ethnopharmacology*.

[13] Liu X., Du J., Cai J. (2007). Clinical systematic observation of Kangxin capsule curing vascular dementia of senile kidney deficiency and blood stagnation type. *Journal of Ethnopharmacology*.

[14] Meng S., Huo Q., Wang B. (2019). Observation of curative effect of Hengqing I Decoction on vascular dementia. *Journal of integrated traditional Chinese and Western medicine*.

[15] Meng S., Huo Q., Wang B. (2020). Study on the effect of Hengqing I decoction on behavior of vascular dementia rats and its mechanism. *Journal of integrated traditional Chinese and Western medicine*.

[16] Dunn W. B., Broadhurst D., Broadhurst D. (2011). Procedures for large-scale metabolic profiling of serum and plasma using gas chromatography and liquid chromatography coupled to mass spectrometry. *Nature Protocols*.

[17] Want E. J., Wilson I. D., Gika H. (2010). Global metabolic profiling procedures for urine using UPLC-MS. *Nature Protocols*.

[18] Wang J., Christison T. T., Misuno K. (2014). Metabolomic profiling of anionic metabolites in head and neck cancer cells by capillary ion chromatography with Orbitrap mass spectrometry. *Analytical Chemistry*.

[19] Xiao J. F., Zhou B., Ressom H. W. (2012). Metabolite identification and quantitation in LC-MS/MS-based metabolomics. *TRAC Trends in Analytical Chemistry*.

[20] Wiklund S., Johansson E., Sjöström L. (2008). Visualization of GC/TOF-MS-based metabolomics data for identification of biochemically interesting compounds using OPLS class models. *Analytical Chemistry*.

[21] Hair J. F. (2010). *Multivariate Data Analysis*.

[22] Jolliffe I. (2002). *Principal Component Analysis*.

[23] Trygg J., Wold S. (2002). Orthogonal projections to latent structures (O-PLS). *Journal of Chemometrics*.

[24] Xia J., Sinelnikov I. V., Han B., Wishart D. S. (2015). MetaboAnalyst 3.0-making metabolomics more meaningful. *Nucleic Acids Research*.

[25] Gravel R. A., Narang M. A. (2005). Molecular genetics of biotin metabolism: old vitamin, new science. *The Journal of Nutritional Biochemistry*.

[26] Zempleni J. (2005). Uptake, localization, and noncarboxylase roles of biotin. *Annual Review of Nutrition*.

[27] Cooper J. L. (2008). P3-400: biotin deficiency and abnormal pantothenic acid levels in dementia. *Alzheimer’s & Dementia*.

[28] Lynch C. J., Adams S. H. (2014). Branched-chain amino acids in metabolic signalling and insulin resistance. *Nature Reviews Endocrinology*.

[29] Selhub J., Troen A. M. (2016). Sulfur amino acids and atherosclerosis: a role for excess dietary methionine. *Annals of the New York Academy of Sciences*.

[30] Yang A., Zhang H., Sun Y. (2015). High-methionine diets accelerate atherosclerosis by HHcy-mediated FABP4 gene demethylation pathway via DNMT1 in ApoE^−/−^ mice. *FEBS Letters*.

[31] Virtanen J. K., Voutilainen S., Rissanen T. H. (2006). High dietary methionine intake increases the risk of acute coronary events in middle-aged men. *Nutrition, Metabolism, and Cardiovascular Diseases*.

[32] Xu J., Patassini S., Begley P. (2020). Cerebral deficiency of vitamin B5 (d-pantothenic acid; pantothenate) as a potentially-reversible cause of neurodegeneration and dementia in sporadic Alzheimer’s disease. *Biochemical and Biophysical Research Communications*.

[33] Herrmann W., Obeid R. (2007). Biomarkers of folate and vitamin B(12) status in cerebrospinal fluid. *Clinical Chemistry and Laboratory Medicine*.

[34] Wagner C., Koury M. J. (2007). S-Adenosylhomocysteine-a better indicator of vascular disease than homocysteine?. *The American Journal of Clinical Nutrition*.

[35] Cervellati C., Romani A., Seripa D. (2014). Oxidative balance, homocysteine, and uric acid levels in older patients with Late Onset Alzheimer’s Disease or Vascular Dementia. *Journal of the Neurological Sciences*.

[36] de las Rivas B., Marcobal Á., Carrascosa A. V., Muñoz R. (2006). PCR detection of foodborne bacteria producing the biogenic amines histamine, tyramine, putrescine, and cadaverine. *Journal of Food Protection*.

[37] Smart S. J., Kroodsma C. T. (2009). Potential relevance of LaPlace’s law to the vascular permeability induced by vasodilators such as histamine and bradykinin. *Journal of Allergy and Clinical Immunology*.

[38] El-kott A. F., Abd-Lateif A. M., Khalifa H. S. (2020). Kaempferol protects against cadmium chloride-induced hippocampal damage and memory deficits by activation of silent information regulator 1 and inhibition of poly (ADP-Ribose) polymerase-1. *The Science of the Total Environment*.

[39] Xiao Q., Ye T., Wang X. (2021). A network pharmacology-based study on key pharmacological pathways and targets of Qi Fu Yin acting on Alzheimer’s disease. *Experimental Gerontology*.

